# SGLT2 Inhibitor Empagliflozin Modulates Ion Channels in Adult Zebrafish Heart

**DOI:** 10.3390/ijms23179559

**Published:** 2022-08-23

**Authors:** Alexey V. Karpushev, Valeria B. Mikhailova, Ekaterina S. Klimenko, Alexander N. Kulikov, Dmitry Yu. Ivkin, Elena Kaschina, Sergey V. Okovityi

**Affiliations:** 1 Sechenov Institute of Evolutionary Physiology and Biochemistry RAS, 44 Thorez Ave., 194223 Saint Petersburg, Russia; 2 Almazov National Medical Research Centre, 2 Akkuratova St., 197341 Saint Petersburg, Russia; 3 Pavlov First State Medical University of St. Petersburg, 6-8 Ulitsa L’va Tolstovo, 197022 Saint Petersburg, Russia; 4 Saint Petersburg State Chemical Pharmaceutical University, 14, Prof. Popov Str., 197376 Saint Petersburg, Russia; 5 Charité-Universitätsmedizin Berlin, Corporate Member of Freie Universität Berlin and Humboldt-Universität zu Berlin, Institute of Pharmacology, Cardiovascular–Metabolic–Renal (CMR)-Research Center, 10115 Berlin, Germany; 6 DZHK (German Centre for Cardiovascular Research), Partner Site Berlin, 10115 Berlin, Germany

**Keywords:** empagliflozin, delayed rectifier potassium current, cardiac action potential, zebrafish heart

## Abstract

Empagliflozin, an inhibitor of sodium-glucose co-transporter 2 (iSGLT2), improves cardiovascular outcomes in patients with and without diabetes and possesses an antiarrhythmic activity. However, the mechanisms of these protective effects have not been fully elucidated. This study aimed to explore the impact of empagliflozin on ion channel activity and electrophysiological characteristics in the ventricular myocardium. The main cardiac ionic currents (I_Na_, I_CaL_, I_CaT,_ I_Kr_, I_Ks_) and action potentials (APs) were studied in zebrafish. Whole-cell currents were measured using the patch clamp method in the isolated ventricular cardiomyocytes. The conventional sharp glass microelectrode technique was applied for the recording of APs from the ventricular myocardium of the excised heart. Empagliflozin pretreatment compared to the control group enhanced potassium I_Kr_ step current density in the range of testing potentials from 0 to +30 mV, I_Kr_ tail current density in the range of testing potentials from +10 to +70 mV, and I_Ks_ current density in the range of testing potentials from −10 to +20 mV. Moreover, in the ventricular myocardium, empagliflozin pretreatment shortened AP duration APD as shown by reduced APD50 and APD90. Empagliflozin had no influence on sodium (I_Na_) and L- and T-type calcium currents (I_CaL_ and I_CaT_) in zebrafish ventricular cardiomyocytes. Thus, we conclude that empagliflozin increases the rapid and slow components of delayed rectifier K^+^ current (I_Kr_ and I_Ks_). This mechanism could be favorable for cardiac protection.

## 1. Introduction

Despite the great advances in medicine, sudden cardiac death remains a leading cause of mortality and is responsible for more than 60% of all deaths from cardiovascular diseases [1]. Sodium-glucose cotransporter 2 inhibitors (isSGLT2) reduce hospitalizations and death from heart failure (HF) [2,3,4]. The underlying mechanisms of their beneficial effects are being intensively studied.

Recent experimental data provided evidence that isSGLT2 empagliflozin, canagliflozin, and dapagliflozin slowed the progression of heart failure in normoglycemic animals [5,6,7,8], and their effectiveness was comparable with ACE inhibitors [9]. The results of DAPA HF- and EMPEROR-reduced randomized clinical trials demonstrated the beneficial effects of isSGLT2 in non-diabetic patients with heart failure [10,11]. Moreover, isSGLT2 exerts cardiorenal protection that co-insides with antiarrhythmic effects [12,13,14]. For instance, dapagliflozin decreased the incidence of reported episodes of atrial fibrillation and atrial flutter adverse events in high-risk patients with type 2 diabetes mellitus [12]. Moreover, a recent meta-analysis of 34 randomized trials with 63,166 patients demonstrated that isSGLT2 is are associated with significantly reduced risks of incident atrial arrhythmias and sudden cardiac death in patients with T2DM [15].

Cardioprotective effects of isSGLT2 in the context of anti-arrhythmias may be attributed to reduced fibrosis and decreased left ventricular hypertrophy [16] as well as to reduced sympathetic activity [17,18]. Recently, several important cellular mechanisms of action of isSGLT2 have been identified, e.g., anti-oxidative and improvement of the cardiac metabolome [19], increased energy production from glucose, ketone bodies, and fatty acid oxidation [20,21], enhanced mitochondrial respiratory capacity [22], anti-inflammation [23], and anti-proteolysis [24]. These mechanisms may contribute to fibroblast activation-related electrical remodeling or the function of different cardiac ion channels.

Importantly, the effects of empagliflozin on the late Na^+^ current [25,26], L-type Ca^2+^ and Na^+^/Ca^2+^ exchanger (NCX) currents [27] have been recently shown. Moreover, empagliflozin reduced calcium-calmodulin kinase II (CaMKII) activity and CaMKII-dependent SR Ca^2+^ leak [28]. Several recent studies have suggested that iSGLT2 also inhibits cardiac Na^+^/H^+^ exchanger (NHE1) activity and expression. Nevertheless, these data are controversial [29,30,31] and the exact anti-arrhythmic mechanism of action of iSGLT2 remains unclear.

Therefore, this study aimed to investigate the influence of empagliflozin on the main ionic currents in the cardiomyocytes and the action potential (AP) profile in the ventricular myocardium of an isolated heart.

## 2. Results

**Figure 1:**
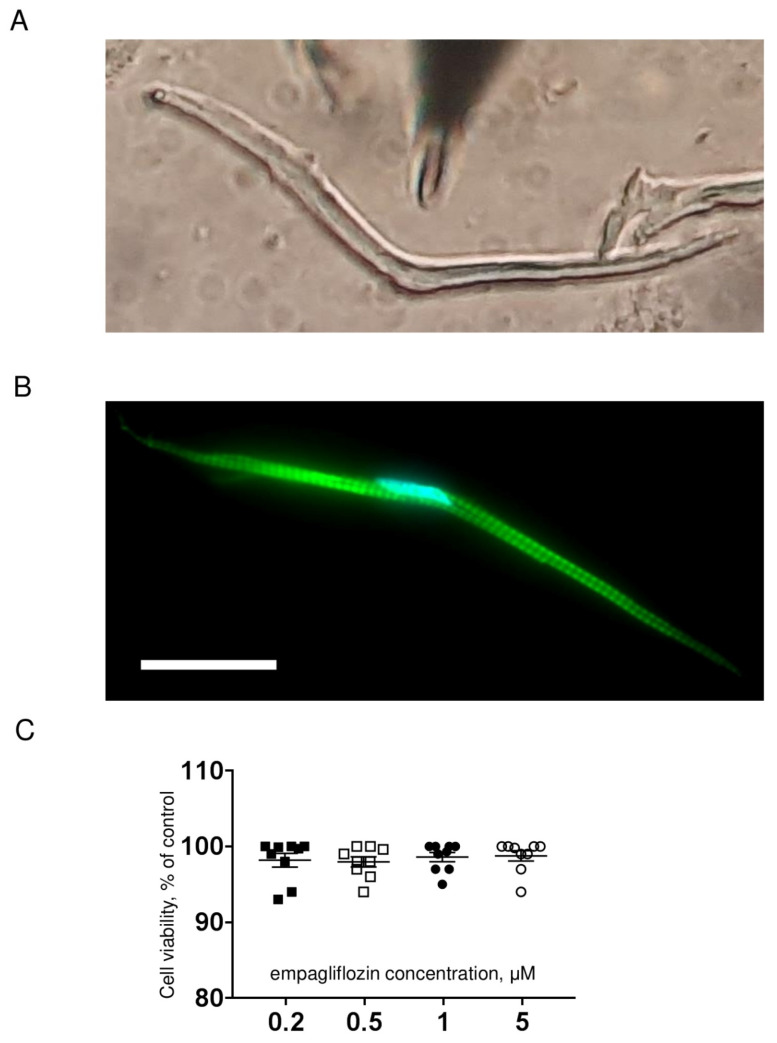
(**A**) Representative image of an isolated zebrafish ventricular cardiomyocyte used for patch clamp experiments. Magnification: 40×. (**B**) Representative image of actin filaments (green) staining using phalloidin in zebrafish cardiomyocytes. Nucleus (blue) was visualized with DAPI. Scale bar is 20 μm. (**C**) Grouped data on MTT test for cardiomyocytes treated with empagliflozin. Data are expressed as % of value obtained for untreated cells.

In our study, we used the zebrafish (*Danio rerio*), a tropical freshwater teleost. Isolated zebrafish ventricular cardiomyocytes appear rod-shaped and quite narrow compared to those of mammals (Figure 1A), as shown previously [32,33]. Phalloidin conjugated with Alexa Fluor 488 was used to visualize the sarcomeric organization of actin, which is clearly seen in the cross-striations (Figure 1B).

To elucidate whether empagliflozin has effects on the cardiac electrical activity we performed experiments to register main ionic currents in freshly isolated cardiomyocytes from zebrafish. The concentration range of empagliflozin was used based on the plasma levels observed clinically [34]. Cardiomyocyte viability was assessed using the MTT assay, the results of which are presented in Figure 1C. No significant differences in cell viability were determined between the control and empagliflozin-treated groups.

**Figure 2:**
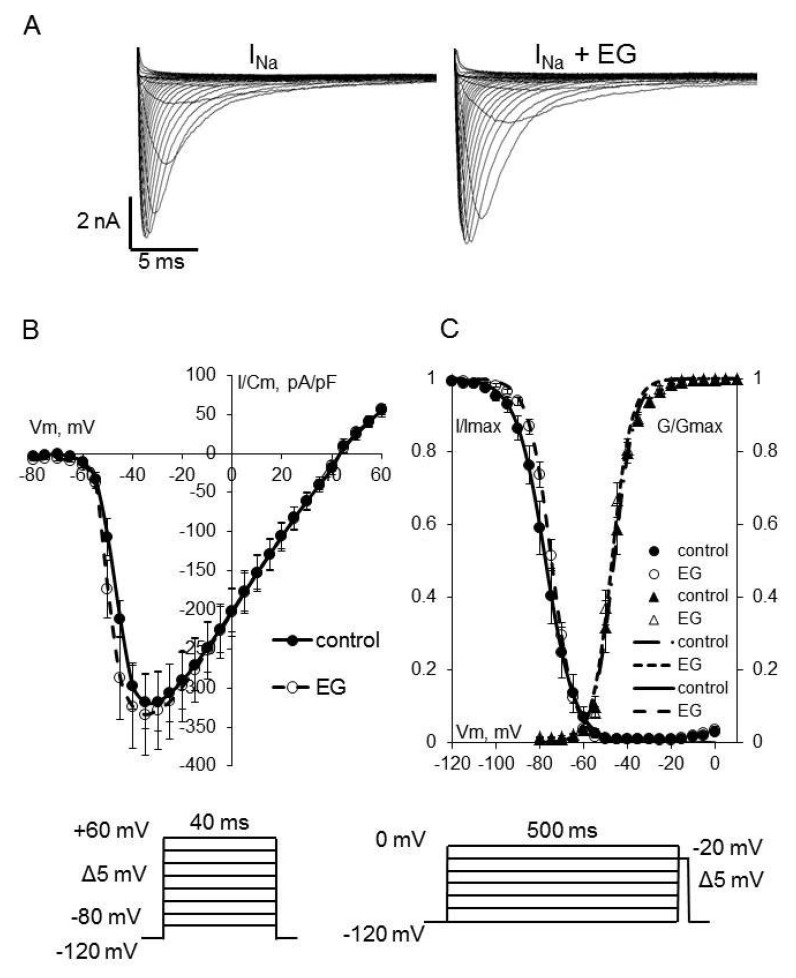
(**A**) Representative whole–cell current traces of I_Na_ in freshly isolated ventricular cardiomyocytes from zebrafish in control (left) and after 2 h incubation with 5 μM empagliflozin (right). (**B**) The current density–voltage relationship of the I_Na_ in control (*n* = 13) and in empagliflozin–treated (*n* = 14) cardiomyocytes. (**C**) The voltage dependence of steady–state activation and inactivation. The solid and dash lines show least–squares fits to the Boltzmann function. Voltage clamp protocols used to determine activation and inactivation characteristics are shown at the bottom.

**Figure 3:**
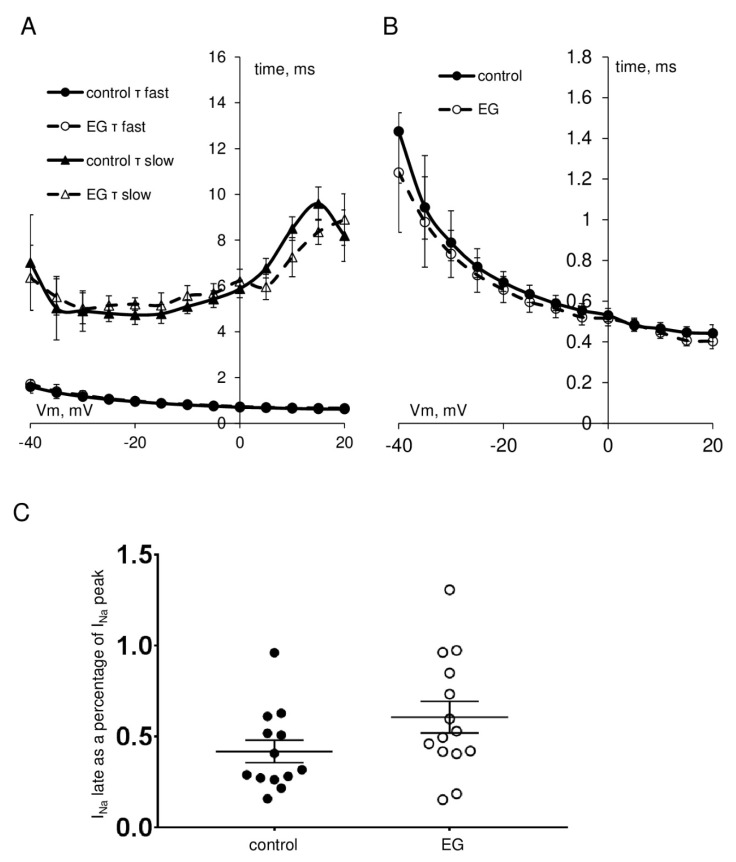
(**A**) The voltage dependence of the inactivation fast (triangles) and slow (circles) time constants τ_fast_, τ_slow_ of I_Na_ in control (filled) and after 2 h incubation with 5 μM empagliflozin (open) cardiomyocytes. (**B**) The voltage dependence of the time to peak of I_Na_. (**C**) Grouped data on I_Na_ late current as a percentage of I_Na_ peak current in control and after 2 h incubation with 5 μM empagliflozin.

**Figure 4:**
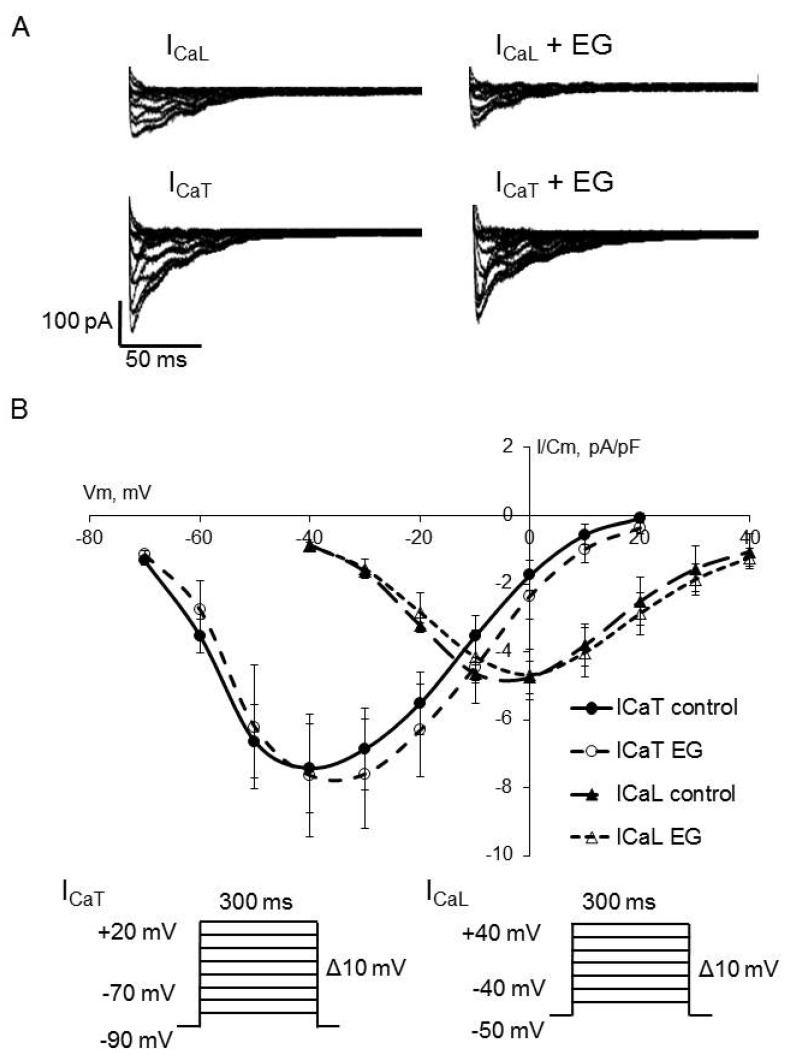
(**A**) Representative whole–cell current traces of I_Ca_ in freshly isolated ventricular cardiomyocytes from zebrafish in control (left) and after 2 h incubation with 5 μM empagliflozin (right). (**B**) The current density–voltage relationship of the I_Ca_ in control (*n* = 14) and in empagliflozin–treated (*n* = 14) cardiomyocytes. Voltage clamp protocols used to estimate I_CaT_ and I_CaL_ are shown at the bottom.

As shown in Figure 2, Figure 3 and Figure 4, the extended incubation of ventricular cardiomyocytes for 2 h in the presence of empagliflozin at the concentration of 5 μM had no effect on I_Na_, I_CaL_, and I_CaT_. Analysis of the current-voltage characteristics of I_Na_, I_CaL_, and I_CaT_ and the parameters of the voltage-dependence of activation and inactivation revealed no significant differences between the control and empagliflozin-treated groups. Table 1 summarizes the biophysical characteristics of examined ionic currents.

**Figure 5:**
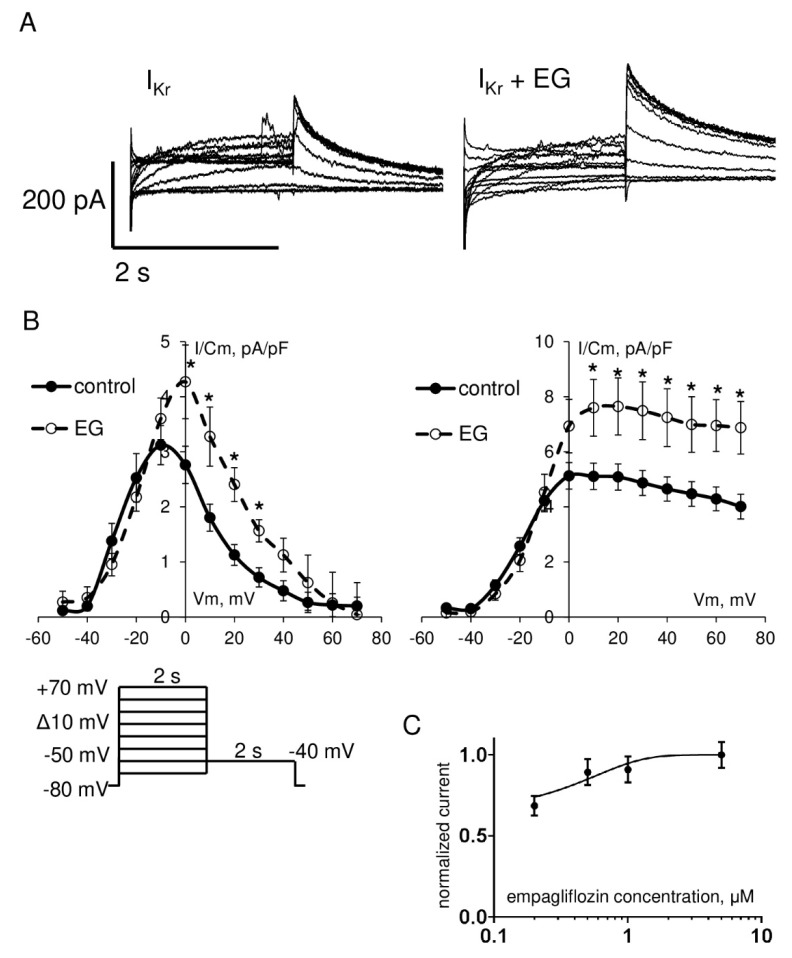
(**A**) Representative whole-cell current traces of I_Kr_ in freshly isolated ventricular cardiomyocytes from zebrafish in control (left) and after 2 h incubation with 5 μM empagliflozin (right). (**B**) The current density–voltage relationship of the I_Kr_ step (left) and tail (right) current in control (*n* = 18) and in empagliflozin–treated (*n* = 19) cardiomyocytes. * *p* < 0.05 obtained by Student’s *t*-test. Voltage clamp protocols used to estimate I_Kr_ are shown at the bottom. (**C**) Concentration–response curve for empagliflozin effect on the I_Kr_ tail current density at +10 mV. The solid line shows least-squares fit to the Hill function.

**Figure 6:**
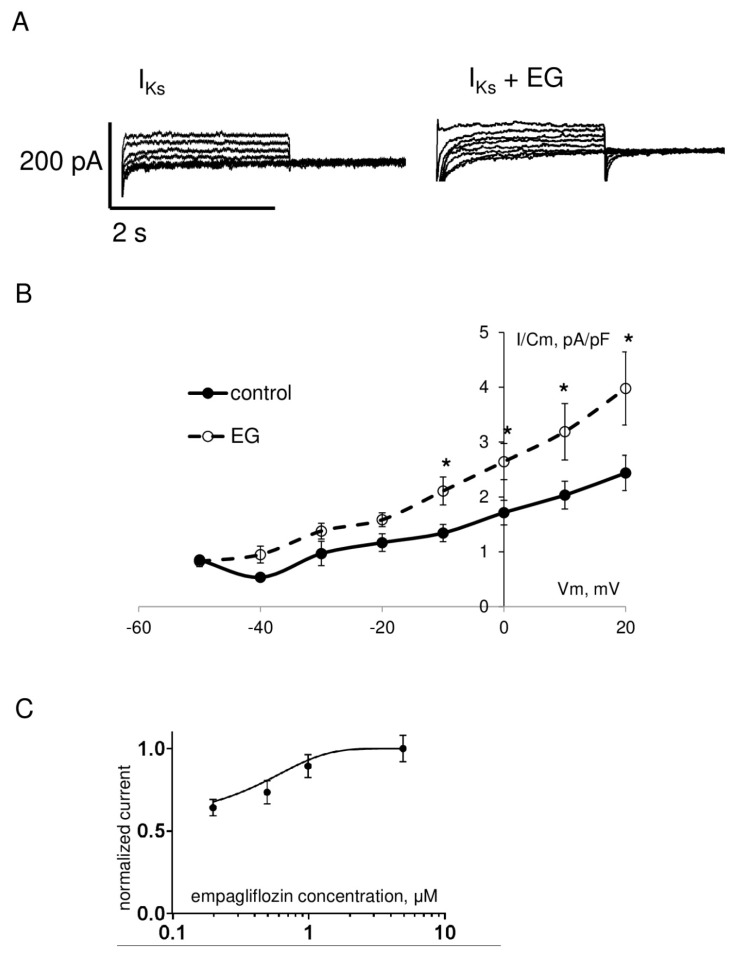
(**A**) Representative whole–cell current traces of I_Ks_ in freshly isolated ventricular cardiomyocytes from zebrafish in control (left) and after 2 h incubation with 5 μM empagliflozin (right). (**B**) The current density–voltage relationship of the I_Ks_ in control (*n* = 18) and in empagliflozin–treated (*n* = 19) cardiomyocytes. * *p* < 0.05 obtained by Student’s *t*-test. (**C**) Concentration–response curve for empagliflozin effect on the I_Ks_ current density at +20 mV. The solid line shows least–squares fit to the Hill function.

However, in contrast to the aforementioned currents, functional analysis of the rapid component of delayed rectifier potassium current I_Kr_ exhibited a significant increase in the amplitude of the step and tail currents. As shown in Figure 5 and Table 1, I_Kr_ step current density in the range of testing potentials from 0 to +30 mV and I_Kr_ tail current density in the range of testing potentials from +10 to +70 mV in cardiomyocytes after empagliflozin pretreatment was significantly enhanced compared to the control group. Analysis of the slow component of delayed rectifier potassium current I_Ks_ revealed a significant increase in the amplitude in the range of testing potentials from −10 to +20 mV in the empagliflozin-treated group (Figure 6). The effect of empagliflozin on the I_Kr_ tail and I_Ks_ current density had a concentration-dependent manner in the range from 0.2 to 5 μM with a half maximal effective concentration (EC_50_) of 0.56 μM and 0.76 μM, respectively.

**Figure 7:**
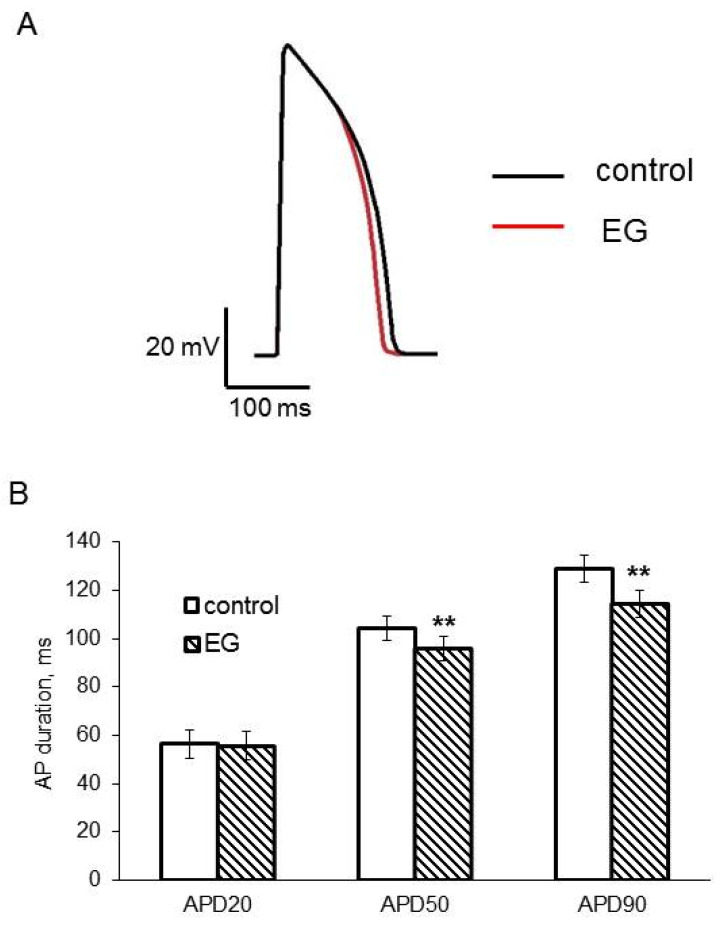
(**A**) Representative recording of ventricular AP from ex vivo zebrafish heart. Black line shows AP in control and red in 5 μM empagliflozin–treated heart. (**B**) Bar graphs of mean results for APD20, APD50, and APD90 in control (*n* = 5) and empagliflozin–treated (*n* = 5) heart. ** *p* < 0.01 obtained by paired Student’s *t*-test.

Since I_Kr_ is one of the major repolarizing currents in zebrafish hearts and an increase in the I_Kr_ current density can result in a change in AP duration, our next step was to explore the AP profile in the ventricular myocardium of an isolated heart. We found, as expected, that the perfusion of the excised heart for 2 h with 5 μM empagliflozin-containing buffer solution significantly reduced APD50 and APD90, but did not affect other AP parameters (Figure 7 and Table 2).

## 3. Discussion

Recently, SGLT2 inhibitors have come into the focus of research due to the cardioprotective effects demonstrated by the EMPA-REG OUTCOME [2]. Dramatic cardiac benefits include the reduced rate of death from cardiovascular causes and the reduction in heart failure hospitalization in people with and without diabetes [10,35]. Despite a growing number of studies investigating the cardiovascular effects of empagliflozin, the underlying mechanisms are still not fully understood and research results are sometimes contradictory [29,36].

The present study was designed to determine the potential effects on the main ionic currents responsible for AP generation in the model object, zebrafish. Zebrafish are widely used as an adequate vertebrate model of human cardiac function due to the presence of a similar set of ionic currents responsible for the AP in cardiomyocytes. For example, while the use of small mammals such as mice and rats can be limited due to the little expression of delayed rectifier potassium current [37] the advantage of using zebrafish is the presence of both rapid and slow components of this current [38]. Zebrafish have similarities with humans in resting membrane potential, AP amplitude, and shape, in particular in the presence of a clear plateau phase [39]. The similarity with mammals is the involvement of I_Na_ in the AP upstroke and I_CaL_ in the plateau phase. Although a fast phase-1 repolarization is not present in zebrafish AP by reason of the absence of the transient outward current I_To_.

A number of studies have already shown the restorative effects of empagliflozin on the increased by some impacts late Na^+^ current [25,26], on the disturbed L-type Ca^2+^ and Na^+^/Ca^2+^ exchanger (NCX) currents in the ventricular myocytes of diabetes mellitus rats [27]. There are controversial data regarding the inhibition of Na^+^/H^+^ exchanger activity by empagliflozin [29,30,31]. In this study, we demonstrate for the first time that empagliflozin affects the rapid and slow components of delayed rectifier potassium current I_Kr_ and I_Ks_. It was revealed significant increase in the amplitude of the I_Kr_ step and tail and I_Ks_ currents in empagliflozin-treated cardiomyocytes. However, we failed to detect any effects on I_Na_, I_CaL_, and I_CaT_.

In the human heart, the I_Kr_ current is conducted by the hERG channel, also known as K_V_11.1 or KCNH2 [40]. I_Kr_ is essential for proper electrical activity in the heart. I_Kr_ is crucially important for determining the cardiac AP duration due to effective control of repolarization [41]. Reduction in I_Kr_ produced by either direct channel block or inhibition of trafficking results in prolonged AP duration that is linked to an increased risk for Torsade de Pointes and, as a consequence, sudden cardiac death [42]. The pore-forming subunit of the I_Ks_ channel, known as KvLQT1 or Kv7.1, is encoded by the *KCNQ1* gene [40]. Reduction in current densities due to loss-of-function *KCNQ1* mutations or a reduction in repolarization reserve during β-adrenergic stimulation is thought to underlie long QT syndrome phenotypes with increasing susceptibility to arrhythmia [43]. Thus, drugs that increase hERG or KvLQT1 activity might have potential antiarrhythmic effects.

Our experiments in the recording of APs from ventricular myocardium have shown a significant reduction in APD50 and APD90 in empagliflozin-treated zebrafish hearts. These data, as expected, show good agreement with those of I_K_ outward current enhancement.

It has been reported that the expression of the hERG channel is significantly downregulated in diabetic hearts due to high-glucose-induced inhibition of channel trafficking, and this downregulation is a critical contributor to the slowing of repolarization [44]. Studies performed using various animal models have reported a decrease in I_Kr_ and I_Ks_ current along with a prolonged QT interval in diabetic dog and rabbit hearts [45]. It is also well established that a reduction in the expression of K_V_ channels in hypertrophied and failing myocardium can result in AP prolongation, which is known to be a pro-arrhythmogenic substrate [46]. Thus, the obtained results allow speculation that the gain of function effect of empagliflozin on I_K_ outward current might be considered as a mechanism of cardioprotective action of the drug. However, when trying to extrapolate the results of our research to humans, the temperature sensitivity of delayed rectifier potassium current should be taken into account [47,48]. The recordings of ionic currents were performed at +28 °C, within the range of physiological temperatures for zebrafish. Moreover, it is noteworthy that I_Kr_ is produced predominantly by a channel encoded by the zebrafish ortholog to the mammalian *KCNH6* gene [49]. These facts should determine objectives of the further study on the effects of empagliflozin.

It should be noted that in some previous studies SGLT2 has not been detected in cardiomyocytes and the heart [50,51,52]. On the other hand, Kwong-Man Ng and coworkers reported SGLT2 expression in hiPSC-derived cardiomyocytes and human heart tissue and they showed that high glucose culture significantly increased SGLT1 and SGLT2 expression in cardiomyocytes [53]. Therefore, what is the pathway of the empagliflozin effect remains a question to be solved. Molecular modeling of empagliflozin docking has shown that the drug has binding affinities to a region in Na_V_1.5 that is a binding site for known sodium channel inhibitors [25]. However, it seems more likely indirect effect, through the activation of signaling cascades. Empagliflozin has been shown to induce vasodilation in the rabbit aorta by activating protein kinase G PKG and K_V_ channels [54]. Empagliflozin reduces the activity of Ca^2+^/calmodulin-dependent kinase II CaMKII in mouse and human failing ventricular myocytes [28]. This makes it possible to assume the presence of signaling pathways mediating empagliflozin effects on I_K_.

In conclusion, results from this study revealed the following key observations: (1) empagliflozin increases I_Kr_ and I_Ks_ currents and has no effects on I_Na_, I_CaL_, and I_CaT_ in zebrafish ventricular cardiomyocytes, (2) empagliflozin shortens AP duration in ventricular myocardium. Summing up the aforementioned, suppose that the cardioprotective effect of the SGLT2 inhibitor may be attributed to the upregulation effect on I_K_ outward current.

## 4. Materials and Methods

### 4.1. Isolation of Ventricular Cardiomyocytes

All animal handling was performed in accordance with the Helsinki convention. One-year-old wild-type zebrafish were used in the experiments.

Ventricular cardiomyocytes were obtained from the heart by enzymatic dissociation. The fish were killed by decapitation. The heart was rapidly excised. A cannula, blunted syringe needle 32 gauge, was introduced through the aortic bulb of the isolated heart for retrograde perfusion for 10–15 min with a Ca^2+^-free solution of the following composition (in mM): 100 NaCl, 10 KCl, 1.2 KH_2_PO_4_, 4 MgSO_4_, 10 HEPES, 50 taurine, 20 glucose, pH 6.9 (adjusted with KOH at room temperature). Then the heart was perfused for 25–30 min with the same solution containing proteolytic enzymes: 0.7 mg/mL collagenase type IA (Sigma-Aldrich, St. Louis, MO, USA); 0.6 mg/mL trypsin, type IX (Sigma-Aldrich, St. Louis, MO, USA), and 1 mg/mL bovine serum albumin (DIA-M, Moscow, Russia). All perfusion was carried out at room temperature, trypsin was used only to obtain cells for registration of Na^+^ current. After the end of perfusion, the atrium was removed and ventricular myocardium was destroyed mechanically (by cutting with surgical scissors and pipetting) to isolate individual cells. Cardiomyocytes were stored in the Ca^2+^-free solution at +4 °C for no more than 8 h.

### 4.2. Cell Viability Assay

Cell viability was determined using 3-(4,5-dimethylthiaz-ol-2-yl)-2,5-diphenyltetrazolium bromide MTT (Sigma-Aldrich, St. Louis, MO, USA). Cardiomyocytes were plated in 96-well plates at 1000 cells per well. Cells were exposed to empagliflozin in the concentration range from 0.2 to 5 μM of empagliflozin for 2 h at +28 °C. All groups were repeated in triplicate and were repeated in three independent experiments. After the treatment, MTT solution was added to the final concentration of 0.5 mg/mL for incubation at +28 °C for 4 h. Then DMSO was added to dissolve formazan crystals. Finally, the absorbance was measured by microplate reader CLARIOstar^®^ Plus (BMG LABTECH, Ortenberg, Germany) at 570 nm. The absorbance reading at 630 nm was used as a reference and was subtracted from the 570-nm absorbance reading. The percentage of living cells was calculated using the following formula:

% of living cells = 100% × [(sample absorbance − blank absorbance)/(control absorbance − blank absorbance)],(1)

where blank absorbance is the absorbance in wells with the buffer solution without cells and control absorbance is the absorbance in wells with untreated cells.

### 4.3. Actin Fluorescent Staining

Cardiomyocytes plated on gelatin-coated coverslips were fixed with 4% paraformaldehyde in PBS for 15 min. After the fixation, cells were soaked with PBS three times for 5 min and then permeabilized with 0.05% Triton X-100 in PBS for 5 min at room temperature. Subsequently, the cells were again washed with PBS twice for 5 min. To visualize actin filaments, the cells were incubated with phalloidin conjugated with Alexa Fluor 488 (Thermo Fisher Scientific, Waltham, MA, USA) for 40 min at room temperature and analyzed under a fluorescence microscope Axio Observer Z1 (Carl Zeiss, Oberkochen, Germany) after counterstaining of nuclei with 4′,6-diamidino-2-phenylindole (DAPI). Images were obtained at a magnification of ×63.

### 4.4. Recording of Ionic Currents

The whole-cell voltage clamp recordings of ionic currents were performed in the freshly isolated ventricular myocytes at +28 °C, which is the standard temperature for zebrafish maintenance in the lab [55] and within the range of physiological temperatures for zebrafish populations reported in the wild [56]. Each control or empagliflozin-treated group consisted of 13–19 cardiomyocytes, 3–5 cells per fish. Data acquisition was performed with amplifier Axopatch 200B and Clampfit software, version 10.3 (Molecular Devices, San Jose, CA, USA). The ionic currents were acquired at 20–50 kHz and low-pass filtered at 5 kHz using the analog-to-digital interface Digidata 1440A acquisition system (Molecular Devices, San Jose, CA, USA). All pulse protocols were applied more than 5 min after membrane rupture. Patch pipettes of 2.5–3.5 MΩ resistance were pulled from the borosilicate glass B150-110-10 (Sutter Instrument, Novato, CA, USA) with a puller P-1000 (Sutter Instrument, Novato, CA, USA). The pipette and cell capacities and access resistance were completely compensated. The series resistance was compensated by 85–90%.

Na^+^ current I_Na_ was recorded in the bath solution contained in mM: 150 NaCl, 3 KCl, 1.8 CaCl_2_, 1.2 MgCl_2_, 10 HEPES, 10 glucose, pH 7.6 (adjusted with NaOH at room temperature). The pipette solution contained in mM: 5 NaCl, 130 CsCl, 1 MgCl_2_, 5 EGTA, 5 HEPES, 5 MgATP, pH 7.2 (adjusted with CsOH). Ca^2+^ and K^+^ currents, I_Ca_ and I_Kr_, were blocked with 10μM nifedipine and 2 μM E-4031 (Tocris, Bristol, UK) added to the external solution.

I_Na_ was elicited from the holding potential of −120 mV with 40 ms depolarizing voltage steps from −80 to +60 mV in 5 mV increment at the frequency of 1 Hz. The current density, I_Na_ normalized to the cell membrane capacitance, was plotted against the voltage steps. Sodium conductance G_Na_ was determined using the equation

G_Na_ = I_Na_/(V − Vrev), (2)

where V is the voltage step, and Vrev is the reversal potential of I_Na_ calculated by a linear extrapolation of peak I_Na_ in the range of depolarization potentials from +10 to +40 mV. The voltage dependence of steady-state activation of I_Na_ was estimated by normalized G_Na_/G_Na max_ plotted against voltage steps and fitted by the Boltzmann equation

G_Na_/G_Na max_ = 1 − 1/(1 + exp^((V − V1/2)/k)^), (3)

where G_Na max_ is the maximal sodium conductance, V_1/2_ is the potential of half-maximal activation of I_Na_, and k is the curve slope factor.

The voltage dependence of steady-state inactivation of I_Na_ was estimated using a double-step protocol with 40 ms testing steps to −20 mV following a conditioning 500 ms prepulse ranging from −120 mV to 0 mV in 5 mV step increment. Normalized I_Na_/I_Na max_ elicited by the testing steps was plotted against the voltage of conditioning prepulse and fitted by the Boltzmann equation.

The time to peak was analyzed as a measure of activation kinetics. Time constants of inactivation were obtained by fitting the decaying phase of current trace with a biexponential equation:

I_t_/I_max_ = A_fast_ × (1 + exp^(−t/τ fast)^) + A_slow_ × (1 + exp^(−t/τ slow)^), (4)

where A_fast_ and A_slow_ are the fractions of fast and slow inactivating components, respectively, and τ fast and τ slow are their time constants. I_Na_ late current was measured at 100 ms after I_Na_ peak at −35 mV and the data are presented as a percentage of I_Na_ peak current.

I_Ca_ was recorded in the bath solution contained in mM: 130 NaCl, 5 CsCl, 2 CaCl_2_, 1 MgCl_2_, 5 Na-pyruvate, 10 HEPES, 10 glucose, pH 7.4 (adjusted with NaOH at room temperature). The pipette solution contained in mM: 130 CsCl, 1 MgCl_2_, 0.345 CaCl_2_, 5 EGTA, 10 HEPES, 5 MgATP, 15 TEA-Cl, pH 7.2 (adjusted with CsOH). I_Na_ and I_Kr_ were blocked with 2 μM tetrodotoxin TTX and 2 μM E-4031 (Tocris, Bristol, UK) added to the external solution.

The total I_Ca_, including I_CaT_ and I_CaL_, was elicited from the holding potential of −90 mV with 300 ms depolarizing voltage steps from −70 to +20 mV in 10 mV increment. I_CaL_ was recorded at depolarization in the range from −40 to +40 mV following the 300 ms step of depolarization up to −50 mV. I_CaT_ was obtained as the difference current between these two protocols.

Delayed rectifier potassium current I_K_ was recorded in the bath solution contained in mM: 150 NaCl, 5.4 KCl, 1.8 CaCl_2_, 1.2 MgCl_2_, 10 HEPES, 10 glucose, pH 7.6 (adjusted with NaOH at room temperature). The pipette solution contained in mM: 140 KCl, 1 MgCl_2_, 5 EGTA, 10 HEPES, 4 MgATP, 0.03 Na_2_GTP, pH 7.2 (adjusted with KOH). I_Na_, I_Ca_, and I_K1_ were blocked with 2 μM TTX, 10 μM nifedipine, (Tocris, Bristol, UK), and 2 mM BaCl_2_ added to the external solution.

I_K_ was elicited by a double-pulse protocol from the holding potential of −80 mV. Initial 2 s depolarization from −50 to +70 mV in 10 mV step increment was followed by 2 s repolarization to −40 mV. Rapid component of delayed rectifier potassium current I_Kr_ was measured as an E-4031-sensitive current. The step and tail current peak amplitude after subtraction of the E-4031-sensitive current was used to assess I_Kr_. Slow component of delayed rectifier potassium current I_Ks_ was obtained as outward step current in the presence of E-4031.

### 4.5. Recording of Action Potentials

APs were recorded from ex vivo heart after cutting off the pacemaker area of the heart (sinoatrial junction). The excised heart preparation consisting of the ventricle and a part of the atrium was pinned on the bottom of the Sylgard-coated chamber and continuously perfused at +28 °C with oxygenated solution contained in mM: 150 NaCl, 5.4 KCl, 1.8 CaCl_2_, 1.2 MgCl_2_, 10 HEPES, 10 glucose, pH 7.4 (adjusted with NaOH). The continuous pacing at 2 Hz frequency was performed. After an hour of equilibration in experimental conditions, the recording of electrical activity was started. The conventional sharp glass microelectrodes technique was used for intracellular recording of APs in ventricular myocardium. The microelectrodes of 20–40 MΩ resistance were filled with 3 M KCl and connected to a high input impedance amplifier model 1600 (A-M Systems, Sequim, WA, USA). The signal was digitized and recorded using PowerGraph 3.3 (DI-Soft, Moscow, Russia) and analyzed using Mini Analysis 3.0 software (Synaptosoft, Fort Lee, NJ, USA). AP duration at 20%, 50%, and 90% of repolarization (APD20, APD50, and APD90, respectively), AP amplitude, and AP upstroke velocity (dV/dt) were determined during offline analysis. Drug was added to the perfusion solution from concentrated stock solutions to yield the final concentration.

### 4.6. Empagliflozin Treatment

To elucidate empagliflozin effects on the main ionic currents in freshly isolated zebrafish cardiomyocytes, cells were incubated for 2 h in the presence of various concentrations of empagliflozin. To determine empagliflozin effects on the AP parameters, the isolated heart was perfused for 2 h with empagliflozin-containing oxygenated buffer solution. Incubation and perfusion were carried out at +28 °C. Further, the recordings of ionic currents or AP were performed in the presence of empagliflozin in the buffer solutions.

### 4.7. Statistical Analysis

Data are presented as mean values ± standard errors (SEM). After checking the normality of the distribution of data obtained by ionic currents recording, statistical comparisons were made using Student’s *t*-test. AP parameters measured in zebrafish heart before and after application of 5 μM empagliflozin were compared using paired Student’s *t*-test. Results with *p* < 0.05 were considered to be statistically significant.

## Figures and Tables

**Table 1:** Biophysical characteristics of I_Na_, I_CaL_, I_CaT_, and I_Kr_ in ventricular cardiomyocytes from zebrafish. Values are represented as mean ± SEM. * *p* < 0.05 obtained by Student’s *t*-test.

	Control	*n*	Empagliflozin	*n*
I_Na_ peak current density at −35 mV, pA/pF	−317.9 ± 34.6	13	−334.4 ± 37.4	14
I_Na_ steady-state activation, V_1/2_, mV k, mV/e-fold	−46.5 ± 1.1 3.7 ± 0.2	13	−48.3 ± 0.9 3.9 ± 0.3	14
I_Na_ steady-state inactivation, V_1/2_, mV k, mV/e-fold	−77.3 ± 2.0 5.1 ± 0.2	13	−76.1 ± 0.9 5.0 ± 0.1	14
I_Na_ late current, % of I_Na_ peak current	0.42 ± 0.09	13	0.61 ± 0.10	14
I_CaL_ peak current density at 0 mV, pA/pF	−4.8 ± 0.7	14	−4.7 ± 0.7	14
I_CaT_ peak current density at −40 mV, pA/pF, ms	−7.4 ± 1.3	14	−7.6 ± 1.8	14
I_CaL_ steady-state activation, V_1/2_, mV k, mV/e-fold	−19.3 ± 1.6 7.7 ± 0.3	14	−16.0 ± 1.7 7.9 ± 0.5	14
I_CaT_ steady-state activation, V_1/2_, mV k, mV/e-fold	−50.2 ± 2.2 9.5 ± 1.3	14	−46.6 ± 2.8 9.1 ± 1.2	14
I_Kr_ peak step current density at 0 mV, pA/pF	2.8 ± 0.3	18	* 4.2 ± 0.7	19
I_Kr_ peak tail current density at +10 mV, pA/pF	5.1 ± 0.4	18	* 7.6 ± 1.0	19
I_Ks_ peak current density at +20 mV, pA/pF	2.4 ± 0.3	18	* 4.0 ± 0.7	19

**Table 2:** Amplitude and time parameters of the AP in ventricular myocardium of isolated zebrafish heart. Values are represented as mean ± SEM. ** *p* < 0.01 obtained by paired Student’s *t*-test.

	Control *n* = 5	Empagliflozin *n* = 5
AP amplitude, mV	82.8 ± 3.0	81.0 ± 2.7
AP upstroke velocity, mV/ms	11.2 ± 0.9	13.8 ± 2.0
APD20, ms	56.5 ± 5.9	55.8 ± 5.9
APD50, ms	104.3 ± 5.0	** 95.7 ± 5.0
APD90, ms	128.8 ± 5.5	** 114.5 ± 5.8

## Data Availability

The data presented in this study are available on request from the corresponding author.

## Abbreviations

AP	Action potential
APD20, APD50, and APD90	Action potential duration at 20%, 50%, and 90% of repolarization, respectively
hERG	Human ether-a-go-go related gene
hiPSC	Human induced pluripotent stem cell
I_CaL_	L-type calcium current
I_CaT_	T-type calcium current
I_Na_	Sodium current
I_Kr_	Rapid component of delayed rectifier potassium current
I_Ks_	Slow component of delayed rectifier potassium current
K_V_	Voltage-gated potassium channel
SGLT2	Sodium-glucose co-transporter 2
i(s)SGLT2	Sodium-glucose cotransporter type 2 inhibitor(s)

## References

[1] Adabag A.S., Luepker R.V., Roger V.L., Gersh B.J. (2010). Sudden cardiac death: Epidemiology and risk factors. Nat. Rev. Cardiol..

[2] Zinman B., Wanner C., Lachin J.M., Fitchett D., Bluhmki E., Hantel S., Mattheus M., Devins T., Johansen O.E., Woerle H.J. (2015). Empagliflozin, Cardiovascular Outcomes, and Mortality in Type 2 Diabetes. N. Engl. J. Med..

[3] Neal B., Perkovic V., Mahaffey K.W., de Zeeuw D., Fulcher G., Erondu N., Shaw W., Law G., Desai M., Matthews D.R. (2017). Canagliflozin and Cardiovascular and Renal Events in Type 2 Diabetes. N. Engl. J. Med..

[4] Wiviott S.D., Raz I., Bonaca M.P., Mosenzon O., Kato E.T., Cahn A., Silverman M.G., Zelniker T.A., Kuder J.F., Murphy S.A. (2019). Dapagliflozin and Cardiovascular Outcomes in Type 2 Diabetes. N. Engl. J. Med..

[5] Byrne N.J., Parajuli N., Levasseur J.L., Boisvenue J., Beker D.L., Masson G., Fedak P.W., Verma S., Dyck J.R. (2017). Empagliflozin Prevents Worsening of Cardiac Function in an Experimental Model of Pressure Overload-Induced Heart Failure. JACC Basic Transl. Sci..

[6] Yurista S., Silljé H.H., Oberdorf Maass S.U., Schouten E., Giani M.G.P., Hillebrands J., Van Goor H., Van Veldhuisen D.J., De Boer R.A., Westenbrink B.D. (2019). Sodium-glucose co-transporter 2 inhibition with empagliflozin improves cardiac function in non-diabetic rats with left ventricular dysfunction after myocardial infarction. Eur. J. Heart Fail..

[7] Lim V.G., Bell R.M., Arjun S., Kolatsi-Joannou M., Long D.A., Yellon D.M. (2019). SGLT2 Inhibitor, Canagliflozin, Attenuates Myocardial Infarction in the Diabetic and Nondiabetic Heart. JACC Basic Transl. Sci..

[8] Cappetta D., De Angelis A., Ciuffreda L.P., Coppini R., Cozzolino A., Miccichè A., Dell'Aversana C., D’Amario D., Cianflone E., Scavone C. (2020). Amelioration of diastolic dysfunction by dapagliflozin in a non-diabetic model involves coronary endothelium. Pharmacol. Res..

[9] Krasnova M., Kulikov A., Okovityi S., Ivkin D., Karpov A., Kaschina E., Smirnov A. (2020). Comparative efficacy of empagliflozin and drugs of baseline therapy in post-infarct heart failure in normoglycemic rats. Naunyn Schmiedebergs Arch. Exp. Pathol. Pharmakol..

[10] McMurray J.J.V., Solomon S.D., Inzucchi S.E., Køber L., Kosiborod M.N., Martinez F.A., Ponikowski P., Sabatine M.S., Anand I.S., Bělohlávek J. (2019). Dapagliflozin in Patients with Heart Failure and Reduced Ejection Fraction. N. Engl. J. Med..

[11] Packer M., Anker S.D., Butler J., Filippatos G., Pocock S.J., Carson P., Januzzi J., Verma S., Tsutsui H., Brueckmann M. (2020). Cardiovascular and Renal Outcomes with Empagliflozin in Heart Failure. N. Engl. J. Med..

[12] Zelniker T.A., Braunwald E. (2020). Mechanisms of Cardiorenal Effects of Sodium-Glucose Cotransporter 2 Inhibitors. J. Am. Coll. Cardiol..

[13] Okunrintemi V., Mishriky B.M., Powell J.R., Cummings D.M. (2020). Sodium glucose co transporter 2 inhibitors and atrial fibrillation in the cardiovascular and renal outcome trials. Diabetes Obes. Metab..

[14] Li H.-L., Lip G.Y.H., Feng Q., Fei Y., Tse Y.-K., Wu M.-Z., Ren Q.-W., Tse H.-F., Cheung B.-M.Y., Yiu K.-H. (2021). Sodium-glucose cotransporter 2 inhibitors (SGLT2i) and cardiac arrhythmias: A systematic review and meta-analysis. Cardiovasc. Diabetol..

[15] Fernandes G.C., Fernandes A., Cardoso R., Penalver J., Knijnik L., Mitrani R.D., Myerburg R.J., Goldberger J.J. (2021). Association of SGLT2 inhibitors with arrhythmias and sudden cardiac death in patients with type 2 diabetes or heart failure: A meta-analysis of 34 randomized controlled trials. Hearth Rhythm.

[16] Santos-Gallego C.G., Requena-Ibanez J.A., Antonio R.S., Garcia-Ropero A., Ishikawa K., Watanabe S., Picatoste B., Vargas-Delgado A.P., Flores-Umanzor E.J., Sanz J. (2020). Empagliflozin Ameliorates Diastolic Dysfunction and Left Ventricular Fibrosis/Stiffness in Nondiabetic Heart Failure. JACC Cardiovasc. Imaging.

[17] Matthews V.B., Elliot R.H., Rudnicka C., Hricova J., Herat L., Schlaich M. (2017). Role of the sympathetic nervous system in regulation of the sodium glucose cotransporter 2. J. Hypertens..

[18] Wan N., Rahman A., Hitomi H., Nishiyama A. (2018). The Effects of Sodium-Glucose Cotransporter 2 Inhibitors on Sympathetic Nervous Activity. Front. Endocrinol..

[19] Oshima H., Miki T., Kuno A., Mizuno M., Sato T., Tanno M., Yano T., Nakata K., Kimura Y., Abe K. (2018). Empagliflozin, an SGLT2 Inhibitor, Reduced the Mortality Rate after Acute Myocardial Infarction with Modification of Cardiac Metabolomes and Antioxidants in Diabetic Rats. J. Pharmacol. Exp. Ther..

[20] Santos-Gallego C.G., Requena-Ibanez J.A., Antonio R.S., Ishikawa K., Watanabe S., Picatoste B., Flores E., Garcia-Ropero A., Sanz J., Hajjar R.J. (2019). Empagliflozin Ameliorates Adverse Left Ventricular Remodeling in Nondiabetic Heart Failure by Enhancing Myocardial Energetics. J. Am. Coll. Cardiol..

[21] Verma S., McMurray J.J.V. (2018). SGLT2 inhibitors and mechanisms of cardiovascular benefit: A state-of-the-art review. Diabetologia.

[22] Seefeldt J.M., Lassen T.R., Hjortbak M.V., Jespersen N.R., Kvist F., Hansen J., Bøtker H.E. (2021). Cardioprotective effects of empagliflozin after ischemia and reperfusion in rats. Sci. Rep..

[23] Byrne N.J., Matsumura N., Maayah Z.H., Ferdaoussi M., Takahara S., Darwesh A.M., Levasseur J.L., Jahng J.W.S., Vos D., Parajuli N. (2020). Empagliflozin Blunts Worsening Cardiac Dysfunction Associated With Reduced NLRP3 (Nucleotide-Binding Domain-Like Receptor Protein 3) Inflammasome Activation in Heart Failure. Circ. Heart Fail..

[24] Hirata Y., Nomura K., Senga Y., Okada Y., Kobayashi K., Okamoto S., Minokoshi Y., Imamura M., Takeda S., Hosooka T. (2019). Hyperglycemia induces skeletal muscle atrophy via a WWP1/KLF15 axis. JCI Insight.

[25] Philippaert K., Kalyaanamoorthy S., Fatehi M., Long W., Soni S., Byrne N.J., Barr A., Singh J., Wong J., Palechuk T. (2021). Cardiac Late Sodium Channel Current Is a Molecular Target for the Sodium/Glucose Cotransporter 2 Inhibitor Empagliflozin. Circulation.

[26] Hegyi B., Hernandez J.M., Shen E.Y., Habibi N.R., Bossuyt J., Bers D.M. (2022). Empagliflozin Reverses Late Na + Current Enhancement and Cardiomyocyte Proarrhythmia in a Translational Murine Model of Heart Failure with Preserved Ejection Fraction. Circulation.

[27] Lee T.-I., Chen Y.-C., Lin Y.-K., Chung C.-C., Lu Y.-Y., Kao Y.-H., Chen Y.-J. (2019). Empagliflozin Attenuates Myocardial Sodium and Calcium Dysregulation and Reverses Cardiac Remodeling in Streptozotocin-Induced Diabetic Rats. Int. J. Mol. Sci..

[28] Mustroph J., Wagemann O., Lücht C.M., Trum M., Hammer K., Sag C.M., Lebek S., Tarnowski D., Reinders J., Perbellini F. (2018). Empagliflozin reduces Ca/calmodulin-dependent kinase II activity in isolated ventricular cardiomyocytes. ESC Heart Fail..

[29] Chung Y.J., Park K.C., Tokar S., Eykyn T.R., Fuller W., Pavlovic D., Swietach P., Shattock M.J. (2020). Off-target effects of sodium-glucose co-transporter 2 blockers: Empagliflozin does not inhibit Na+/H+ exchanger-1 or lower [Na+]i in the heart. Cardiovasc. Res..

[30] Baartscheer A., Schumacher C.A., Wust R.C., Fiolet J.W.T., Stienen G., Coronel R., Zuurbier C.J. (2016). Empagliflozin decreases myocardial cytoplasmic Na+ through inhibition of the cardiac Na+/H+ exchanger in rats and rabbits. Diabetologia.

[31] Uthman L., Baartscheer A., Bleijlevens B., Schumacher C.A., Fiolet J.W.T., Koeman A., Jancev M., Hollmann M.W., Weber N.C., Coronel R. (2017). Class effects of SGLT2 inhibitors in mouse cardiomyocytes and hearts: Inhibition of Na+/H+ exchanger, lowering of cytosolic Na+ and vasodilation. Diabetologia.

[32] Brette F., Luxan G., Cros C., Dixey H., Wilson C., Shiels H.A. (2008). Characterization of isolated ventricular myocytes from adult zebrafish (Danio rerio). Biochem. Biophys. Res. Commun..

[33] Verkerk A.O., Remme C.A. (2012). Zebrafish: A novel research tool for cardiac (patho)electrophysiology and ion channel disorders. Front. Physiol..

[34] Scheen A.J. (2014). Pharmacokinetic and Pharmacodynamic Profile of Empagliflozin, a Sodium Glucose Co-Transporter 2 Inhibitor. Clin. Pharmacokinet..

[35] Zannad F., Ferreira J.P., Pocock S.J., Anker S.D., Butler J., Filippatos G., Brueckmann M., Ofstad A.P., Pfarr E., Jamal W. (2020). SGLT2 inhibitors in patients with heart failure with reduced ejection fraction: A meta-analysis of the EMPEROR-Reduced and DAPA-HF trials. Lancet.

[36] Uthman L., Baartscheer A., Schumacher C.A., Fiolet J.W.T., Kuschma M.C., Hollmann M.W., Coronel R., Weber N.C., Zuurbier C.J. (2018). Direct Cardiac Actions of Sodium Glucose Cotransporter 2 Inhibitors Target Pathogenic Mechanisms Underlying Heart Failure in Diabetic Patients. Front. Physiol..

[37] Sutanto H., Heijman J. (2022). Integrative Computational Modeling of Cardiomyocyte Calcium Handling and Cardiac Arrhythmias: Current Status and Future Challenges. Cells.

[38] Abramochkin D.V., Hassinen M., Vornanen M. (2018). Transcripts of Kv7.1 and MinK channels and slow delayed rectifier K+ current (IKs) are expressed in zebrafish (Danio rerio) heart. Pflügers Arch. Eur. J. Physiol..

[39] Gauvrit S., Bossaer J., Lee J., Collins M.M. (2022). Modeling Human Cardiac Arrhythmias: Insights from Zebrafish. J. Cardiovasc. Dev. Dis..

[40] Chen L., Sampson K.J., Kass R.S. (2016). Cardiac Delayed Rectifier Potassium Channels in Health and Disease. Card. Electrophysiol. Clin..

[41] Sanguinetti M.C., Tristani-Firouzi M. (2006). hERG potassium channels and cardiac arrhythmia. Nature.

[42] Brown A.M. (2007). hERG Assay, QT Liability, and Sudden Cardiac Death. Cardiac Safety of Noncardiac Drugs.

[43] Bohnen M.S., Peng G., Robey S.H., Terrenoire C., Iyer V., Sampson K.J., Kass R.S. (2017). Molecular Pathophysiology of Congenital Long QT Syndrome. Physiol. Rev..

[44] Shi Y.-Q., Yan M., Liu L.-R., Zhang X., Wang X., Geng H.-Z., Zhao X., Li B.-X. (2015). High Glucose Represses hERG K+ Channel Expression through Trafficking Inhibition. Cell. Physiol. Biochem..

[45] Ozturk N., Uslu S., Ozdemir S. (2021). Diabetes-induced changes in cardiac voltage-gated ion channels. World J. Diabetes.

[46] Mustroph J., Maier L.S., Wagner S. (2014). CaMKII regulation of cardiac K channels. Front. Pharmacol..

[47] Vandenberg J.I., Varghese A., Lu Y., Bursill J.A., Mahaut-Smith M.P., Huang C.L.-H. (2006). Temperature dependence of human ether-à-go-go-related gene K+ currents. Am. J. Physiol. Physiol..

[48] Walsh K.B., Begenisich T.B., Kass R.S. (1989). Beta-adrenergic modulation of cardiac ion channels. Differential temperature sensitivity of potassium and calcium currents. J. Gen. Physiol..

[49] Vornanen M., Hassinen M. (2016). Zebrafish heart as a model for human cardiac electrophysiology. Channels.

[50] Vrhovac I., Eror D.B., Klessen D., Burger C., Breljak D., Kraus O., Radović N., Jadrijević S., Aleksic I., Walles T. (2014). Localizations of Na+-d-glucose cotransporters SGLT1 and SGLT2 in human kidney and of SGLT1 in human small intestine, liver, lung, and heart. Pflügers Arch. Eur. J. Physiol..

[51] Chen J., Williams S., Ho S., Loraine H., Hagan D., Whaley J.M., Feder J.N. (2010). Quantitative PCR tissue expression profiling of the human SGLT2 gene and related family members. Diabetes Ther..

[52] Zhou L., Cryan E.V., D'Andrea M.R., Belkowski S., Conway B.R., Demarest K.T. (2003). Human cardiomyocytes express high level of Na+/glucose cotransporter 1 (SGLT1). J. Cell. Biochem..

[53] Ng K.-M., Lau Y.-M., Dhandhania V., Cai Z.-J., Lee Y.-K., Lai W.-H., Tse H.-F., Siu C.-W. (2018). Empagliflozin Ammeliorates High Glucose Induced-Cardiac Dysfuntion in Human iPSC-Derived Cardiomyocytes. Sci. Rep..

[54] Seo M.S., Jung H.S., An J.R., Kang M., Heo R., Li H., Han E.-T., Yang S.-R., Cho E.-H., Bae Y.M. (2020). Empagliflozin dilates the rabbit aorta by activating PKG and voltage-dependent K+ channels. Toxicol. Appl. Pharmacol..

[55] Westerfield M. (2000). The Zebrafish Book. A Guide for the Laboratory Use of Zebrafish (Danio rerio).

[56] Engeszer R.E., Patterson L.B., Rao A.A., Parichy D.M. (2007). Zebrafish in The Wild: A Review of Natural History And New Notes from The Field. Zebrafish.

